# The contribution of PM_2.5_ to cardiovascular disease in China

**DOI:** 10.1007/s11356-020-09996-3

**Published:** 2020-07-20

**Authors:** Shuqi Zhang, Michael N. Routledge

**Affiliations:** 1 School of Public Health, Fudan University, Shanghai, 200032 China; 2 Leeds Institute of Cardiovascular & Metabolic Medicine, University of Leeds, Leeds, LS2 9JT UK; 3 School of Food and Biological Engineering, Jiangsu University, Zhenjiang, China

**Keywords:** PM_2.5_, Air pollution, Cardiovascular mortality, Ischemic heart disease, Stroke, China

## Abstract

China is experiencing rapid urbanization and industrialization with correspondingly high levels of air pollution. Although the harm of PM_2.5_ has been long reported, it is only quite recently that there is increasing concern in China for its possible adverse health effects on cardiovascular disease. We reviewed the epidemiologic evidence of potential health effects of PM_2.5_ on cardiovascular disease reported from recent studies in China (2013 onwards). There is clear evidence for the contribution of PM_2.5_ to cardiovascular outcomes, including mortality, ischemic heart disease, and stroke from studies based in various regions in China. This evidence adds to the global evidence that PM_2.5_ contributes to adverse cardiovascular health risk and highlights the need for improved air quality in China.

## Introduction

Cardiovascular disease (CVD) is a group of diseases of multifactorial origin that involve damage to the heart and/or vascular system. Contributing factors include hypertension, dyslipidemia, diabetes, and obesity (GBD 2017 Risk Factor Collaborators 2018; Cercato and Fonseca 2019). Cigarette smoking is another major contributor, as a result of damage to the vasculature that results from the regular inhalation of a cocktail of noxious chemicals, including those in gaseous form and those carried on particulate matter (Conklin et al. 2019). It is not surprising, therefore, that inhaled particulates from other sources, including industrial emissions, heating fuels, and traffic exhaust emissions, can also contribute to risk. There is growing evidence for an association between long-term exposure to fine particulate matter and cardiovascular mortality (Requia et al. 2018; Rajagopalan et al. 2018; Yuan et al. 2019). In addition, people with existing cardiovascular disease, from whatever origin, are at risk of acute and potentially fatal cardiovascular events such as heart attack and stroke. A common factor in such events is the formation or rupture of a blood clot that can block blood vessels leading to the death of heart muscle or neural cells (Robertson and Miller 2018). Consequently, it is important to understand the role of air borne particulates in both the long-term and short-term effects on populations undergoing high levels of exposure as well as considering that some people are particularly susceptible to short-term peaks in exposure. The aging population and acceleration of urbanization augment the prevalence of cardiovascular disease in China. According to statistics from the China CDC, cardiovascular diseases were the top cause of death in 2016, which was 45.50% in rural areas and 43.16% in urban areas with a continuous increasing trend (National Center for Cardiovascular Diseases 2017).

China is a large and rapidly developing country with great variations in socioeconomic status among its population. Rapid economic growth has led to increased industrial pollution, and huge urban development and urban wealth has been accompanied by increases in traffic emissions. Consequently, many types of particulate matter, from industrial processes and traffic emissions, co-exist, with variation in distribution of specific types of particulates in different regions. Understandably, many studies have been carried out in the mega cities such as Beijing, Shanghai, and Guangzhou but there have been an increasing number of studies in recent years around China. In this review, we focus on the impact of PM_2.5_ on cardiovascular health, especially as reported in studies published since 2013, a period of rapidly increasing research in this area.

## Search strategy

We searched the databases PubMed and Web of Science for epidemiological literature on the cardiovascular effect of PM_2.5_ published between 2013 and 2019. Combinations of the following keywords were used: (1) particulate, particulate matter, PM, air pollution; (2) cardiovascular disease, heart attack, stroke, cerebrovascular disease, cardiovascular mortality, CVD; (3) China. The search strategies identified the studies conducted in China that examined the adverse effects of exposure to PM_2.5_ on cardiovascular health. The inclusion criteria we used for selection were as follows: (1) The subjects of epidemiological studies were all Chinese population. (2) Original studies expressed quantitative relationships between PM_2.5_ and health outcomes, e.g., relative risk, odds ratios, excess risk (ER), or hazard ratio. (3) The health outcomes involved CVD mortality, IHD, or cerebrovascular disease. For the selected studies, the title, authors, location, publication year, study period, study design, number of events/controls, measure of PM_2.5_ increase, health outcomes, statistical methods, and specific risk estimates were extracted and entered into Microsoft Excel database. The search results were grouped into 3 sections according to health outcomes including cardiovascular end points, IHD end points, and cerebrovascular end points.

## PM_2.5_ and cardiovascular mortality in China

PM_2.5_ are particles of mean aerodynamic diameter below 2.5 μm and consist of a carbon core associated with various inorganic pollutants (e.g., sulfates, nitrates, metals) and organic combustion products (Newby et al. 2014). Particles of this size, especially those below 0.1 μm, can reach deep into the respiratory system, and some may be able to enter the bloodstream (Mills et al. 2009; Pan et al. 2016). Thus, the toxic metals and organic chemicals they carry may potentially be delivered to the circulatory system or trigger an inflammatory response in the lungs that impacts on the cardiovascular system.

As seen in other countries, high PM_2.5_ concentrations are associated with cardiovascular mortality in China (Yin et al. 2017; Table 1). In a prospective study, average PM_2.5_ concentrations between 2000 and 2005 were estimated from a variety of data across 45 disease surveillance points in China, from which nearly 222,000 men were enrolled in a National Cohort of 40-year-old men in 1990–1991. This study covered a wide geographical distribution of the population, with a range of health and lifestyle data and follow-up from death certificates. PM_2.5_ exposures were estimated using a combination of satellite-derived and chemical transport model estimates, calibrated to surface measurements. It was found that for every 10 μg/m^3^ increase in PM_2.5_, the hazard ratio (HR) was 1.09 (95% CI 1.08–1.10) and dose response curves were calculated showing increased risk of cardiovascular death at average PM_2.5_ concentrations above about 20 μg/m^3^.

**Table 1:** Cardiovascular mortality with increased risk associated with PM_2.5_ exposure

Location, etc.	Time period	Outcome	Measure of PM_2.5_ increase	Excess risk or hazard ratio (HR) (95% CI)	Reference
Short-term exposure					
Guangzhou 104,756 cases	2009–2011	CVD mortality	Previous 1–3 days, IQR	6.11% (1.76–10.64%)	Lin et al. (2016a)
Guangzhou 46,850 cases	2013–2015	a) CVD mortality b) CBD (cerebrovascular)	Hourly peak at lag03 day	a) 1.15% (0.67–1.63%) b) 1.09% (0.27–1.91%)	Lin et al. (2017)
30 counties 379,133 cases	2013–2015	a) CVD mortality b) AMI	Same-day exposure, average increase per 10 μg/m^3^	a) 0.12% (0.001–0.25%) b) 0.42% (0.03–0.81%)	Chen et al. (2018)
Shenyang 62,159 cases	2013–2016	CVD mortality	Same-day exposure, average increase per 10 μg/m^3^	0.4% (0.22–0.59%)	Liu et al. (2019)
Beijing Outdoor workers 136,922 cases	2010–2012	CVD mortality	a) 7 days > 75 μg/m^3^ b) 9 days > 105 μg/m^3^	a) 30% (13–50%) b) 53% (29–52%)	Wang et al. (2017)
Yanbian Korean Autonomous Prefecture 16,365 cases (9029 ethnic Hans, 7336 ethnic Koreans)	2015–2016	CVD mortality	Lag1, average increase per 10 μg/m^3^	2.6% (2.5–2.8%)	Zhang et al. (2018a)
Shenzhen 14,537 cases	2013–2015	a) CVD mortality b) CBD (cerebrovascular)	a) Lag5 and lag02, rose by 10 μg/m^3^ b) Lag4 and lag04, rose by 10 μg/m^3^	a) 1.50% (0.51–2.50%); 2.09% (0.79–3.41%) b) 2.09% (2.28–3.92%); 3.08% (0.68–5.53%)	Cai et al. (2018)
7 cities	2013–2015	CVD mortality	Lag01, average increase per 10 μg/m^3^	0.315% (0.133–0.497%)	Liang et al. (2017)
Guangdong 131,765 cases	2013–2015	a) CVD mortality b) AMI	Daily exceedance concentration hours (DECH); average increase of 500 μg/m^3^ h at lag03	a) 4.55% (3.59–5.52%) b) 3% (1.13–4.9%)	Lin et al. (2018)
Average annual exposure					
45 locations, 222,000 40-year-old men	2000–2005	CVD mortality	Average increase per 10 μg/m^3^	HR = 1.09 (95% CI 1.08–1.10)	Yin et al. (2017)

In Guangzhou, southern China, Lin et al. (2016a) reported a significant impact of PM_10_, PM_2.5_, and PM_1_ concentrations in the previous 1–3 days on cardiovascular mortality for the period 2009–2011. The excess risk was 6.1% (95% CI 1.76–10.64%) for PM_10_, 6.11% (95% CI 1.76–10.64%) for PM_2.5_, and 6.48% (95% CI 2.10–11.06%) for PM_1_ per IQR increase in the particulates. Importantly, the analysis controlled for numerous potential confounders, including the weather and influenza epidemics. For PM_2.5_, further breakdown of results by chemical constituents showed that organic carbon, elemental carbon, sulfate, nitrate, and ammonium were significantly associated with cardiovascular mortality. Focusing on short time increases in pollution, the same group (Lin et al. 2017) found significant associations between hourly peak concentrations of PM_2.5_ and cardiovascular mortality, particularly from ischemic heart diseases (IHD) and cerebrovascular diseases (CBD). This highlights the potential for acute peak levels of pollution to impact on cardiovascular mortality, although no significant association was observed for acute myocardial infarction (AMI). Hourly peak concentration of PM_2.5_ was defined in this study as the maximum concentration of 24-h PM_2.5_ values within a given day.

More recently, Chen et al. (2018) have reported an increased risk in cardiovascular mortality with short-term increased PM_2.5_ concentrations from a large multicenter study including data covering the period 2013–2015, collected from 30 counties across northern, eastern, and southwestern China. Controlling for 24-h temperature and relative humidity, this study found that for a 10-μg/m^3^ increase in PM_2.5_, there was a 0.12% (95% CI 0.001–0.25%) increase in CVD mortality and a 0.42% (95% CI 0.03–0.81%) increase in AMI on the same day. The authors noted that while this confirms the impact of short-term exposure to PM_2.5_, the magnitude of the effect was lower than that seen in similar multicenter studies in the USA and Europe. They also reported a higher susceptibility of effects among people aged 65–74.

Analyzing data from 2013 to 2016 from the heavily polluted city of Shenyang, the city with the largest population in northeast China, Liu et al. (2019) reported an increase in daily CVD mortality of 0.4% (95% CI 0.22–0.59%) for each 10 μg/m^3^ increase in PM_2.5_ on the same day. They found that CVD mortality was 2–8 times higher during the warmer months, even though pollution increases during the cold winters when there was more heating used. The authors suggested that a possible reason for this may be that people go out less often in the winter or that higher temperatures outdoors exacerbate the effects of the particulates.

The studies described above provide further evidence from China that short-term peaks in PM_2.5_ exposure are associated with increased cardiovascular mortality. But in cities such as Beijing, there is often prolonged exposure to high levels of this and other pollutants. Wang et al. (2017) studied CVD mortality data throughout 2010, 2011 and 2012 in relation to extended periods of high PM_2.5_ (at least > 75 μg/m^3^) as recorded at the US Embassy monitoring station in central Beijing. They found that for periods of more than 7 days with levels continuously > 75 μg/m^3^, the excess risk of cardiovascular deaths increased. At 7 days continuous concentrations > 75 μg/m^3^, the excess risk for outdoor workers was estimated to be 30% (95% CI 13–50%), rising to 53% (95% CI 29–52%) for 9 days continuously > 105 μg/m^3^. At levels of PM_2.5_ > 115 μg/m^3^, for which prolonged continuous exposure events were rarer, the effect on CVD mortality was already seen at 6 days continuous exposure. In addition to the observed impact on outdoor workers, increased excess risk was observed for single and illiterate subgroups in the study.

China is a vast country with different climatic zones, and both local variations in pollution sources and meteorological conditions may influence the contribution of particulates to health burden. Lanzhou is an industrialized city on the banks of the Yellow River in the semi-arid Gansu Province in the northwest of China. Cardiovascular mortality peaks in the cold, dry winter. Using the data collected for daily pollution levels during 2014–2017, Wu et al. (2019) examined cardiovascular mortality in relation to airborne pollution and found an association between higher PM_2.5_ levels and cerebrovascular deaths, with high PM_10_ being also associated with ischemic heart disease mortality. During this period, the average annual levels of PM_2.5_ were 60–70 μg/m^3^, which is two times the annual mean national air quality standard II (35 μg/m^3^). Overall, the health impacts of PM_2.5_ were found to be greater than PM_10_. A study in Yanbian Korean Autonomous Prefecture, a region with lower average particulate levels, just below the 35 μg/m^3^ standard, also found that higher PM_2.5_ concentrations were associated with higher incidence of cardiovascular and cerebrovascular deaths (Zhang et al. 2018a). For each 10 μg/m^3^ increase in lag1 day concentration of PM_2.5_, there was a 2.6% (95% CI 2.5–2.8%) increase in cardiovascular mortality in this population. The authors point out that this effect is more severe than for some studies reported in more polluted areas of China such as Beijing or Shenyang, suggesting that the dose response curve may show higher responses at lower ends of the curve. Another city with a relatively low annual average PM_2.5_ level (at 35 μg/m^3^) is Shenzhen in southern Guangdong Province. Cai et al. (2018) found that for the years 2013–2015, there were also significant effects of increased PM_2.5_ concentrations on cardiovascular mortality, ischemic heart disease mortality, and cerebrovascular mortality. It is clear therefore that PM_2.5_ concentrations pose a cardiovascular health risk at levels below official air quality standards.

There are different approaches to measuring particulate concentrations, including mean daily concentration (used by Wu et al. 2019; Zhang et al. 2018a, b; Cai et al. 2018; Liang et al. 2017) and hourly peak concentration (used by Lin et al. 2017). A novel measure of measuring concentration of PM_2.5_ was proposed by Lin et al. (2018) to study the effects of high particulate concentrations on cardiovascular mortality. Daily exceedance concentration hour (DECH) is an indicator designed to focus on the length of time levels exceed a threshold, selected as 25 μg/m^3^, and calculated as the number of hours of exceedance multiplied by the level of exceedance in micrograms per cubic meter. Using this indicator in models studying the contribution of PM_2.5_ levels on cardiovascular deaths in six cities from the subtropical Pearl River Delta region, Lin et al. (2018) reported a greater effect of the particulates compared with other studies. An increase of 500 μg/m^3^ h at lag03 was associated with an increase of 4.55% (95% CI 3.59–5.52%) in cardiovascular mortality, a 4.45% (95% CI 2.81–6.12%) increase in ischemic heart disease deaths, and a 3% (95% CI 1.13–4.9%) increase in AMI related deaths. The authors suggest that the daily mean concentration could be underestimating the effects of high air pollution, possibly because the DECH takes into account the peak exceedances that may have a greater impact on triggering cardiovascular events in susceptible individuals. The authors argue for the need to ensure peak exceedances are reduced by interventions that target mean exposure levels.

The fact that multiple pollutants (including PM_2.5_, PM_10_, NO_2_, SO_2_) contribute to the health impacts of air pollution means that the role of various pollutants on their own or together can be complex to determine. Tong et al. (2018) used a statistical learning method based on Bayesian kernel machine regression to estimate the contribution of joint effects on CVD deaths in Beijing. They showed that there could be interaction between pollutants enhancing cardiovascular mortality. They also found that the main contributing factor varied depending on the lag period used to determine the moving average concentrations, with PM_2.5_ making the biggest contribution for 0–3- or 0–4-day moving averages but not 0–1- or 0–2-day moving averages.

## PM_2.5_ and ischemic heart disease in China

An association between increased short-term exposure to PM_2.5_ and ischemic heart disease (IHD) has been shown in several studies conducted in both developed and developing countries (Gouveia et al. 2006; Devos et al. 2015). In China, such associations have also been reported (Table 2). A time series analysis in Hong Kong that assessed effects of fine particles on daily emergency cardiovascular hospitalizations (Qiu et al. 2013) found that an IQR increase in lag01 concentration of PM_2.5_ corresponded to 1.75% (95% CI 0.94–2.57%), 2.61% (1.60–3.64%), and 3.47% (1.84–5.12%) increases of emergency hospital admissions for total circulatory diseases, cardiac diseases, and IHD, respectively.

**Table 2:** Ischemic heart disease end points with increased risk associated with PM_2.5_ exposure

Location, etc.	Time period	Outcome	Measure of PM_2.5_ increase	Excess risk (95% CI)	Reference
Short-term exposure					
Hong Kong 612,551 cases	2000–2005	IHD emergency hospital admissions	Lag01, IQR	3.47% (1.84–5.12%)	Qiu et al. (2012)
Beijing 8544IHD (56,221 total cardiovascular ERVs)	2013–2015	IHD emergency room visits (ERVs)	Lag0–1, average increase per 10 μg/m^3^	0.56% (0.16–0.95%)	Xu et al. (2017a)
Beijing 369,469 IHD cases; 53, 247 IHD deaths	2010–2012	a) IHD morbidity b) IHD mortality	Same-day exposure, average increase 10 μg/m^3^	a) 0.27% (0.21–0.33%) b) 0.25% (0.10–0.40%)	Xie et al. (2015)
Shanghai 188,198 cases	2013–2014	IHD hospitalizations	From lag0 to lag7, average increase 10 μg/m^3^	0.25% (0.10–0.39%)	Xu et al. (2017b)
Nationwide (26 large cities) 720,261 cases	2014–2016	IHD hospital admissions	Lag2, IQR	1.7% (1.5–1.9%)	Dai et al. (2018)

In Beijing, Xu et al. (2017a) collected daily counts of cardiovascular hospital emergency room visits (ERVs) from ten large hospitals, in which 56,221 cardiovascular ERVs were included. It was estimated that daily mean PM_2.5_ concentration was 102.1 μg/m^3^ in Beijing in 2015, and every 10 μg/m^3^ increase in PM_2.5_ was associated with a 0.14% (95% CI 0.01–0.27%) increase in cardiovascular ERVs at lag3. For the analysis of cause-specific ERVs, the estimates of percentage change in daily ERVs each 10 μg/m^3^ increase in PM_2.5_ were 0.56% (95% CI 0.16–0.95%) for ischemic heart disease (IHD) at lag0–1, 0.81% (95% CI 0.05–1.57%) for heart rhythm disturbances at lag0–1, and 1.21% (95% CI 0.27–2.15%) for heart failure (HF) at lag0, respectively. In addition, this study suggested that the acute effects of PM_2.5_ on IHD varied by temperature with the effect during high-temperature days being significantly higher than that on low-temperature days.

A time series study in Beijing assessed the relationship between PM_2.5_ concentration and IHD morbidity and mortality based on the data of 369,469 IHD cases and 53, 247 IHD deaths from the Beijing Monitoring System for Cardiovascular Diseases during 2010 to 2012 (Xie et al. 2015). The group estimated a 10-μg/m^3^ increase in PM_2.5_ was associated with a 0.27% (95% CI 0.21–0.33%, *p* < 2.00 × 10^−16^) increase in IHD morbidity and a 0.25% (95% CI 0.10–0.40%, *p* = 1.15 × 10^−3^) increase in IHD mortality on the same day. The study also indicated the dose–response relationships were non-linear, with a steeper dose–response function at lower concentrations and a shallower response at higher concentrations. Such coincidence between the escalation of PM_2.5_ and the increase in IHD morbidity was also demonstrated in Xu et al.’s study (2017b). Using daily IHD hospitalization data and average concentration of PM_2.5_, they found that a 10-μg/m^3^ increment increase of PM_2.5_ was associated with an increase in IHD hospitalizations by 0.57% (95% CI 0.46–0.68%). The study (Xu et al. 2017b) suggested that the results could provide important outlook for public health research and management to prevent the increased risk of IHD due to PM by ways of increasing public health awareness and improving government policies.

Using electronic hospitalization summary reports in 26 large Chinese cities, a multicity case-crossover study (Dai et al. 2018) compared the effects of PM concentrations on IHD in northern China and southern China. As the study showed, PM concentrations were much higher in the north than south. In northern China, an IQR increase in PM_2.5_ concentrations at lag2 days was associated with a 1.8% (95% CI 1.6–2.1%) increase in IHD. Nevertheless, in southern China, negative associations were observed with PM_2.5_ almost at all lag structures indicating that the association is null or negative.

Several recent studies in different parts of China have shown an association between PM_2.5_ and acute myocardial infarctions (AMIs) (see Table 3). In Shanghai, Wang et al. (2016) assessed 972 patients from the emergency medical service documents covering the 2013–2014 in the Pudong District. It was found that the mean urban background levels of PM_2.5_ was associated with an increased risk of AMIs, with an OR (95% CI) of 1.16 (1.03–1.29). In addition, the study suggested that more AMIs occurred during 6:00–18:00 when people were more likely to be outdoors and during November, December, and January when the pollution level was high. The study was consistent with that of Yu et al. (2018) conducted in Guangzhou province, in which it was found that a 10-μg/m^3^ increment in concentrations of PM_2.5_ was associated with an increase of 1.636% (95% CI 0.537–2.740%) in AMI.

**Table 3:** Acute myocardial infarction end points with increased risk associated with PM_2.5_ exposure

Location etc	Time period	Outcome	Measure of PM_2.5_ increase	Excess risk, Hazard Ratio (OR) or Hazard Ratio (HR) (95% CI)	Reference
Short-term exposure					
Guangzhou 5545 cases	2015–2016	Daily MI morbidity	2-day cumulative, average increase 10 μg/m^3^	1.636% (0.537–2.740%)	Yu et al. (2018)
Shanghai 972 cases	2013–2014	AMI morbidity	Lag0–1, average increase 10 μg/m^3^	OR = 1.16 (95% CI 1.03–1.29)	Wang et al. (2016)
Beijing 2749 cases	2014	EDVs for AMI and its subtypes a) AMI b) ST-elevation myocardial infarction (STEMI) c) non-ST-elevation myocardial infarction (NSTEMI)	Lag1, average increase 10 μg/m^3^	a) No association b) 1.05 (1.00–1.11) c) No association	Zhang et al. (2016)
Long-term exposure					
Tianjin 598 patients with AMI	2013–2014	Major adverse cardiovascular events (MACEs)	a) Five groups based on National Air Quality Classification Standard b) Better vs worse air quality	a) HR = 1.622 (95% CI 1.352–1.947) b) HR = 3.255 (95% CI: I 2.008–5.276)	Zang and Qi (2017)

Based on 2749 patients from Chaoyang District, Beijing, hospitalized with AMI in Anzhen Hospital, Zhang et al. (2016) estimated the transient effects of PM_2.5_ on risk of EDVs for AMI, ST-elevation myocardial infarction (STEMI), and non-ST-elevation myocardial infarction (NSTEMI). The result showed that each 10-μg/m^3^ increment of PM_2.5_ concentration (1-day lagged) was associated with an increased risk of EDVs for STEMI (OR 1.05; 95% CI 1.00–1.11), especially in patients aged ≥ 65 years. However, no association of PM_2.5_ concentration with overall AMI or NSTEMI was found. The findings indicated that studies of the association between PM_2.5_ and AMI should consider AMI subtypes, lag times, and individual characteristics.

Zang and Qi (2017) implemented a retrospective analysis of a total of 598 patients in Tianjin who were divided into five groups according to the National Air Quality Classification Standard and then divided into two groups according to better and worse air quality. After 1-year follow-up, the study found that with the increase of the concentration of PM_2.5_, the incidence of major adverse cardiovascular events (MACEs) was higher in patients 1 year after AMI. In the five groups, HR was 1.622 (95% CI 1.352–1.947; *p* = 0.000) and, in the two groups, HR was 3.255 (95% CI 2.008–5.276; *p* = 0.000), suggesting that the poor air quality could worsen the condition of patients.

Beside hospital emergency room/department visits for cardiovascular disease, numbers of out-of-hospital heat attack cases, especially cardiac arrest, manifest the acute effects of PM_2.5_ on people with heart disease. Cardiac arrest is defined as the cessation of cardiac mechanical activity and the subsequent cessation of blood circulation (Mozaffarian et al. 2016). To assess the effects of air pollution on out-of-hospital cardiac arrests (OHCAs), Xia et al. (2017) obtained health data from nationwide emergency medical service database and analyzed 4720 OHCA cases. After adjustment for humidity and temperature, they found that the highest odds ratios of OHCA for a 10-μg/m^3^ increase in PM_2.5_ were observed at lag1 (1.07; 95% CI 1.04–1.10), with significant associations with advanced age (aged ≥ 70 years) (1.09; 95% CI 1.05–1.13) and stroke history (1.11; 95% CI 1.06–1.16), supporting the statement that increased PM_2.5_ exposure contributes to triggering OHCA.

## PM_2.5_ and stroke in China

As a subtype of CVD, stroke risk is adversely impacted by PM_2.5_ (Table 4). The modified health risk of stroke associated with PM_2.5_ and PM_10_ was examined in a case-crossover study in 26 Chinese cities by Liu et al. (2017a). Using hospital admission for stroke, the group examined 278,980 hospital admissions for ischemic stroke and 69,399 for hemorrhagic strokes separately from 2013 to 2015. The results showed an IQR in PM_2.5_ and PM_10_ in northern China corresponded to a 1.0% (95% CI 0.7%, 1.4%) and 0.7% (95% CI, 0.3%, 1.2%) increase in ischemic stroke admissions at lag3 days, respectively, indicating the levels of PM_2.5_ and PM_10_ are significantly associated with increased risk of ischemic stroke and PM_2.5_ has a greater adverse effect on ischemic strokes. The different adverse effects of particular size on stroke have been also illustrated in the study of Lin et al. (2016b) in Guangzhou. The group assessed 9066 stroke deaths and found significant association between hemorrhagic stroke mortality with various PM fractions, including PM_10_, PM_2.5_, and PM_1_. It was found that excess risks (ERs) per IQR increase in lag2 days in PM_10_, PM_2.5_, and PM_1_ were 7.18% (95% CI 1.62–13.05%), 6.62% (95% CI 1.28–12.25%), and 8.74% (95% CI 1.33–16.68%), respectively, and the risk estimate of PM on hemorrhagic stroke mortality was more significant. By analyzing PM_2.5_ chemical constituents, the same group found that organic carbon (OC), elemental carbon (EC), sulfate, nitrate, and ammonium were all significantly associated with stroke mortality.

**Table 4:** Cerebrovascular end points with increased risk associated with PM_2.5_ exposure

Location, etc.	Time period	Outcome	Measure of PM_2.5_ increase	Excess risk, hazard ratio (OR), or hazard ratio (HR) (95% CI)	Reference
Short-term exposure					
Gansu 25,790 cases	2014–2017	Cerebrovascular mortality	Lag4, average increase per 10 μg/m^3^	1.22% (0.11–2.35%)	Wu et al. (2019)
26 cities case-crossover 348,379 cases: IS: 278,980; HS: 69,399	2013–2015	Hospital admissions for stroke	Lag3, IQR	1.0% (0.7–1.4%)	Liu et al. (2017a)
Guanzhou 9066 cases	2007–2011	a) Ischemic stroke b) Hemorrhagic stroke	Lag2, IQR	a) No association b) 6.62% (1.28–12.25%)	Lin et al. (2016b)
Beijing 48,122 cases: IS 32,799; HS 13,051	2014–2016	a) Stroke mortality b) Ischemic stroke c) Hemorrhagic stroke	Average increase per 10 μg/m^3^	a) 0.27% (0.12–0.43%) b) 0.23% (0.04–0.42%) c) 0.37% (0.07–0.67%)	Zhang et al. (2018b)
6 cities 54,236 cases: IS: 31,457 HS: 22,779	2013–2016	Stroke mortality	Lag3, average increase per 10 μg/m^3^	3.07% (2.35–3.79%)	Wang et al. (2018)
Guangdong 95,562 cases	2013–2015	Ischemic stroke	Same-day exposure, IQR	1.027% (1.018–1.037%)	Guo et al. (2017)
172 cities 2,032,667 cases	2014–2016	Hospital stroke admissions	Average increase per 10 μg/m^3^	0.34% (0.2–0.48%)	Tian et al. (2018)
Beijing 63,956 cases	2010–2012	Ischemic stroke	Average increase per 10 μg/m^3^	0.31% (95% CI 0.17–0.45%)	Tian et al. (2017)
Chengdu 84, 535 cases	2013–2015	Stroke mortality	Average increase per 10 μg/m^3^	0.69% (0.01–1.38%)	Zeng et al. (2018)
Nationwide 1356 cases	2013–2015	First stroke	Average increase per 10 μg/m^3^	OR = 1.049 (95% CI 1.038–1.061)	Guan et al. (2018)
Long-term exposure					
Hong Kong 66,820 aged 65+ 6733 cases	1998–2001 to 2010	First stroke	Average increase per 10 μg/m^3^	HR = 1.14 (95% CI 1.02–1.27)	Qiu et al. (2017)
Nationwide 12,291 ischemic stroke patients	2007–2008	Nationwide	Average increase per 10 μg/m^3^	HR = 1.05 (95% CI 1.02–1.09)	Chen et al. (2019)

In Beijing, the capital of China, Zhang et al. (2018b) detected a significant association between short-term increased PM_2.5_ with stroke mortality using daily PM_2.5_ concentration from an ecological study. Each 10-μg/m^3^ increase of PM_2.5_ was associated with an increase of mortality: 0.27% (95% CI 0.12–0.43%) for cerebrovascular diseases, 0.23% (95% CI 0.04–0.42%) for ischemic stroke, and 0.37% (95% CI 0.07–0.67%) for hemorrhagic stroke. It was estimated in the same study that if the annual mass concentration of PM_2.5_ could achieve the standard of WHO Air Quality Guidelines (10 μg/m^3^), around 400 stroke deaths will be avoided per year in Beijing. Different from other studies (Zeng et al. 2018; Tian et al. 2018), Zhang et al. (2018b) did not find evidence indicating that gender or age can modify the effect of PM_2.5_ on stroke in subgroup analysis.

Likewise, exploring data from 2013 to 2016 including daily mean concentration of air pollution and daily data on stroke mortality in six subtropical cities in Guangzhou province, Wang et al. (2018) examined the city-specific acute effects of daily PM_10_, PM_2.5_, and PM_10–2.5_ across different lag days (log0 to log3) and multiday lags (log01, log02, and log03). The analysis of lag-specific associations between particulate matters and stroke mortality demonstrated that the increased stroke mortality at lag03 date was most significant. In addition, the study used attributable mortality (AM) and attributable fraction (AF) as indicators to illustrate the mortality burden based on the estimated effects of particulate matters at lag03, the result of which showed that 5.57% (95% CI 4.21–6.96%) of the overall stroke mortality could be attributable to PM_2.5_. The corresponding ER in total stroke mortality in the six cities was 3.07% (95% CI 2.35–3.79%) for PM_2.5_, and sensitivity analyses showed that adverse effect of PM_2.5_ on ischemic stroke was higher than hemorrhagic stroke.

In recent years, a greater number of studies in China further focused on the health effect of PM_2.5_ on the subtype of stroke, especially ischemic stroke. For example, Guo et al.’s study (2017) in Guangdong, with a total of 95,562 attack admissions for ischemic stroke included in this study, assessed the ischemic stroke risk ratio of the same-day exposure to PM_2.5_, which was estimated to be 1.0270 (95% CI 1.0177–1.0368) with an IQR increase in the concentration level of PM_2.5_ in single-pollutant model. Moreover, based on hospital stroke admissions in 2014–2016 from the national database covering up to 0.28 billion people who received Urban Employee Basic Medical Insurance, Tian et al. (2018) examined the association between air pollution and daily ischemic stroke admission nationwide. A two-stage method was used to estimate the effects of air pollution in specific cities and combine all the estimates of 172 cities in China. The results demonstrated that an increase of 10 μg/m^3^ of PM_2.5_ was associated with a 0.34% (95% CI 0.20–0.48%) increase of hospital admissions for ischemic stroke. After controlling for calendar time, relative humidity, public holidays, and day of the week, further subgroup exploration showed that the effect estimates were greater in cities with lower air pollutant levels and higher air temperatures, as well as in elderly subpopulations. The same study explained that high temperature could consistently enhance the effects of air pollution, while the positive effect modification of long-term air pollutant levels was less consistent, indicating that high temperature might exert greater modifying effects on the associations than long-term air pollution levels.

More recently, the association between short-term changes in PM_2.5_ and first hospital admission for ischemic stroke in Beijing was assessed in 63,956 first hospital admissions for ischemic stroke that were identified from Beijing Medical Claim Data for Employees during 2010 to 2012 (Tian et al. 2017). The exposure–response relationship between PM_2.5_ and admissions for ischemic stroke was approximately linear, with a relatively stable response at lower concentrations (< 100 μg/m^3^) and a steeper response at higher concentrations. A 10-μg/m^3^ increase in the same-day PM_2.5_ concentration was associated with a 0.31% (95% CI 0.17–0.45%, *p* < 1.57 × 10^−5^) increase in the daily admissions for ischemic stroke.

Zeng et al. (2018) studied the impact of haze in Chengdu in the Sichuan basin, during which the atmosphere warms with altitude and cool air gets trapped at lower altitudes due to the local geography. Using environmental and daily morbidity of stroke data (2013–2015), the study estimated the daily mean concentration of PM_2.5_ to be 75.9 μg/m^3^ with 38% of the observed days exceeding the Grade II national standards, 75 μg/m^3^ for 24-h mean levels and concluded that same-day exposure to PM_2.5_ was associated with the onset of stroke. For females, every 10 μg/m^3^ increase of PM_2.5_ contributed to 0.80% percent change of onset; for the group of age less than 65, a 0.78% higher risk increase of PM_2.5_ was observed.

A more specific study on differential susceptibility by Guan et al. (2018) assessed association between individual stroke risk and exposure to PM_2.5_ based on data of 1356 first-ever stroke events derived from the China National Stroke Screening Survey (CNSSS) database (*n* = 1,292,010). A significant association between first-ever stroke and PM_2.5_ was found; for each 10 μg/m^3^ increase of PM_2.5_ exposure, the odds ratio was 1.049 (95% CI 1.038, 1.061). The size of association, varied considerably according to individual-specific characteristics including demographic, lifestyle, and medical history factors, ranging OR = 0.966 (95% CI 0.920–1.013) to 1.145 (95% CI 1.080–1.215) among Chinese adults.

Dong et al. (2018) determined that PM_2.5_ can also moderate associations of stroke risk for other pollutants. To investigate the acute effect of NO_2_ and SO_2_ on IS and IS-related death in five urban districts in Changzhou between 2015 and 2016, they estimated the percentage change (95% CI) in daily IS counts and deaths with an IQR increase in air pollutant levels for different single or multiple lag days in both single-pollutant and two-pollutant models. The study found that for daily IS counts, the estimated effects of NO_2_ and SO_2_ were more significant when adjusted for PM_2.5_ and PM_10_. Young individuals (< 65 years old) had a higher IS mortality risk for PM_2.5_, PM_10_, NO_2_, and CO.

The aforementioned studies suggested that in China, short-term PM_2.5_ exposure is associated with increased stroke mortality and morbidity. In addition, several recent cohort studies in China have indicated the long-term effect of PM_2.5_ on stroke. A cohort study in Hong Kong (Qiu et al. 2017) collected data from 66,820 old people (65+) whose first occurrence of emergency hospital admission for stroke was during 1998–2001 (baseline) and followed up to 2010. A total of 6733 cases of incident stroke, of which 3526 (52.4%) were ischemic and 1175 (17.5%) were hemorrhagic, were identified. The results showed the HR for every 10 μg/m^3^ higher PM_2.5_ concentration was 1.14 (95% CI 1.02–1.27) for all incident stroke. However, long-term PM_2.5_ exposure had different associations according to stroke subtype, with a statistically significant HR (1.21, 95% CI 1.04–1.41) for ischemic stroke and no significant association for hemorrhagic stroke (HR 0.90, 95% CI 0.70–1.17). The effect tended to be greater at older ages for those aged > 70 and was higher for less educated people and current smokers.

Similarly assessing the prolonged effects of air pollution, while different from most studies whose outcome is stroke mortality or hospital admission, a few studies examined the adverse effects of air pollution on survival after stroke. Chen et al. (2019) followed 12,291 ischemic stroke patients for 1 year, and during the process, 1649 deaths were identified. After controlling for potential confounders, significant associations were observed between exposure to PM_2.5_ and incident fatal ischemic stroke, with the corresponding HR of 1.05 (95% CI 1.02–1.09). The authors concluded that pre-stroke exposure to PM_2.5_ in China was associated with increased incident fatal ischemic stroke in the year following an ischemic stroke.

## Discussion

As the concerns over the adverse effects of air pollution on human health increases, more and more relevant studies have been reported worldwide, suggesting that air pollution, especially PM_2.5_ is one of the largest environmental causes of cardiovascular disease and death (Lozano et al. 2012). China, the biggest developing country, was observed to have much higher particular matter levels, which might lead to a more conspicuous relationship between exposure and adverse cardiovascular events. The review of recent studies in China, from 2013 onwards, demonstrated that exposure to PM_2.5_ is indeed associated with adverse cardiovascular health effects, including cardiovascular mortality, stroke, and IHD in China.

Although the evidence for the association between air pollution and CVD is strong and growing in volume, there are currently only a few studies focusing on the role of specific chemical constituents in particulate matter. Exploring the relationship between the elemental composition of PM_2.5_ and cardiovascular disease in emergency department patients, Huang et al. (2017) assessed the correlation between emergency admissions for cerebral hemorrhage, cerebral infarction, TIA (transient ischemic attack), coronary heart disease, and PM_2.5_ concentrations of chemical element compositions and PM_10_ in Changsha, Hunan. The study suggested that concentration rises of nickel, zinc, and lead elements for PM_2.5_ in Changsha city were related to the increase of ED for cerebral hemorrhage. With each additional IQR increase of Ni, Zn, and Pb concentrations in PM_2.5_, the values of OR of cerebral hemorrhage were 1.826 (95% CI 1.031–3.233), 1.568 (95% CI 1.015–2.423), and 1.682 (95% CI 1.010–2.800), respectively. Therefore, future research should focus on identification and quantification of chemical compounds present in ambient air particles for a better understanding of the mechanism by which particulate matter causes health effects. To what degree is it that the concentrations of fine particulate matter themselves or their components contribute to these effects, or do PM_2.5_ act as surrogate markers for other components of air pollution, such as gaseous pollution?

To date, there have been a limited number of studies in China estimating long-term health effects of PM_2.5_. Previously, several studies in other countries have found that the mortality of long-term exposure of PM_2.5_ was ten times higher than that of short-term exposure (Dockery et al. 1993; Pope et al. 2004). It is likely that more data from cohort studies in China will emerge in the coming years, which will likely highlight the impact of high pollution levels in certain regions of China.

Methodological advances are also welcomed as these may help to elucidate the interactions between pollution and specific conditions. Although daily mean concentration is widely used as exposure indicators to estimate PM_2.5_, a study (Guo et al. 2018) showed that daily mean concentrations might insufficiently represent the true exposure level because of the diurnal variations of air pollutants and various human activity patterns, while daytime or rush-hour concentrations may lead to better estimations of acute effect of PM_2.5_. Lin et al. (2018) used DECH to address concerns that the commonly used measurement indicator; daily mean concentration could underestimate the mortality risk. Therefore, the application of advanced methodological approaches to future studies may help to pin down the most significant aspects of air pollution contributing to disease risk, especially for acute events such as heart attack and stroke.

Exposure measurement is notoriously difficult for air pollution studies, with compromises between geographically wide estimates of exposure and sample size needed to be made in may studies. The difference and relationship between measured PM_2.5_ and actual PM_2.5_ need further clarification. The data used by most Chinese studies was obtained from local environmental monitoring stations or the U.S. embassy station, which, on the one hand, may not be totally verified or validated, and, on the other hand, may only represent the general levels of outdoor air pollution rather than the actual PM_2.5_ levels. In addition, the impact of other factors such as socioeconomic traits and meteorological conditions may vary from region to region. Consequently, a suggestion for the improvement of environmental policy is to establish systematic PM_2.5_ monitor network across the country and to consider regional and population variations more carefully. In particular, studies related to PM_2.5_ exposure on cardiovascular health are largely centered in highly urbanized megacities, particularly in Beijing, Shanghai, Guangzhou, or regions with high PM_2.5_ pollution, like Taiyuan. In contrast with urban cities, the number and scope of studies in rural areas or remote areas with relative small population, like Gansu (Wu et al. 2019) and Yanbian Korean Autonomous Prefecture (Zhang et al. 2018a), are still much fewer.

Indoor pollution from domestic heating and cooking, as well as smoking, might also have significant effects on people’s cardiovascular health, so the measurement of indoor PM_2.5_ would help to reduce risk of confounding. Likewise, the PM air quality standards and control measures are based on the report of outdoor air pollution from monitor stations, which might ignore the possible health threat of indoor pollution. So it is recommended that the government should consider the function of indoor PM_2.5_ when making environmental standards and relative protective approaches to minimize these adverse effects; meanwhile, the public should improve awareness of self-protection even when they are indoors.

China has been developing rapidly in recent years. Urbanization, accompanied with increased industrialization, emissions from waste, biomass burning, as well as vehicle exhaust, has led to considerable increase of PM_2.5_ in China during the past several years. By investigating the nexus between urbanization and the air pollution-related health, Liu et al. (2017b) found a 1% increase in urbanization was associated with a 0.32%, 0.14%, and 0.50% increase in PM_2.5_-related mortality of lung cancer, stroke, and ischemic heart disease, indicating that the launch of China’s new national urbanization plan may result in worse PM_2.5_-related health effects. Under these circumstances, some studies accessing the public health benefits of reducing PM_2.5_ in China emphasized the significant of air quality improvement. For instance, Chen et al. (2017) created an annual air quality surface with a land use regression (LUR) model to evaluate avoided cases of mortality and morbidity in Tianjin, assuming the achievement of China’s current air quality daily and annual standards (no. GB3095-2012). It was estimated in the study (Chen et al. 2017) that if the daily average PM_2.5_ was reduced to the daily Class II standard (75 μg/m^3^), the avoided deaths for cardiovascular disease would be 2000 (95% CI 920–3100) per year and the monetary values were 180–4800 million yuan per year in 2015 in Tianjin; if the annual average PM_2.5_ was reduced to the annual Class II standard (35 μg/m^3^), the avoided deaths for cardiovascular disease were 1400 (95% CI 640–2100) and the monetary values were 130 to 3400 million yuan.

To eliminate the concerns over the health effects of PM_2.5_ in general public and obtain the benefit of addressing PM_2.5_ issues, Chinese government must take a series of effective actions. Some improvement of policy-making has been achieved by government and found really helpful in reducing the detrimental impact of air pollution and remedying people’s health state. A time series study (Su et al. 2016) in Beijing observed adverse effects of PM_2.5_ on cardiovascular ERV before Beijing Olympics using polynomial distributed lag (PDL) models and found that an IQR increase in PM_2.5_ was associated with an overall RR of 1.022 (95% CI 0.990–1.057). However, modification occurred on account of some main air pollution control policies taken during the period of Olympics; as the study showed, cardiovascular ERV in association with air pollution increases in the non-Olympics period while no effects were observed during the Olympics period and the difference for PM_2.5_ was statistically significant. In addition, “The Airborne Pollution Prevention and Control Action Plan (2013–2017),” the goal of which is to reduce 25% of PM emissions from 2012 levels by the year 2017, was established by the government to improve air quality through installing emissions-cutting exhaust filters, reducing coal use, tightening vehicle emission standards, etc. (Sheehan et al. 2014). Although air quality daily and annual standards in China (no. GB3095-2012) have played an essential role in the last 7 years since 2012, stricter standards need establishing to improve air quality, such as primary-level standard. The government could decide more appropriate standards by referring to WHO guidelines and the standards used by other countries. For example, in 2013, the EPA (Environmental Protection Agency) in the USA published a new regulation for PM in which the annual primary standards for PM_2.5_ were reduced to 12 μg/m^3^ from the previous criterion of 15 μg/m^3^. And several recent studies (Yin et al. 2017; Ye et al. 2019) exploring the shape of exposure–response relation of PM_2.5_ against cause-specific mortality and morbidity and the saturation point of PM_2.5_ might also provide evidence for standards setting and environmental policy-making. Meanwhile, as has mentioned before, China is a vast county with uneven levels of PM_2.5_; therefore, the standards should be adjusted to better adapt to regional conditions.

Moreover, support from industry and public combined with policy-making is necessary in addressing the challenge from PM_2.5_ air pollution. The industries should enhance social responsibilities to comply with relative guidelines and control the emission of pollution by means of developing new technologies, such as gasoline particular filters. Meanwhile, the public could develop personal awareness to take timely protective measures when necessary and help improve air quality, corresponding to policy requirements. For example, wearing masks and using air purifiers especially in high-polluted areas and when warning saturation point is reported reduce the usage of power-wasting vehicles and burning of biomass for cooking and heating indoors and adopt more environmentally friendly travel and living habits. By working together, government, industry, and the general population can cooperate to reduce hazardous pollution.

## Conclusion

Here, we have summarized findings from an ever increasing number of studies that highlight the adverse impact of PM_2.5_ on cardiovascular health in China. Focusing on one aspect of air pollution, PM_2.5_, which may on the one hand be directly involved as a causative risk factor in at least some end points and on the other hand may be a surrogate for other pollutants contributing to the mechanism of disease, the results of the various studies to date provide strong evidence for the role of ambient air pollution in cardiovascular disease risk. While further studies, in particular on long-term effects of PM_2.5_ exposure, will add to the knowledge base, the existing evidence from China and elsewhere supports the need for a reduction in such pollution in order to reduce the incidence of cardiovascular disease. This is particularly important in China, where recent rapid economic advances have led to high levels of air pollution in many parts of the country.

## References

[CR1] Cai JF, Yu SY, Pei YX, Peng CQ, Liao YX, Liu N, Ji JJ, Cheng JQ (2018). Association between airborne fine particulate matter and residents’ cardiovascular diseases, ischemic heart disease and cerebral vascular disease mortality in areas with lighter air pollution in China. Int J Environ Res Public Health.

[CR2] Cercato C, Fonseca FA (2019). Cardiovascular risk and obesity. Diabetol Metab Syndr.

[CR3] Chen L, Shi MS, Li SH, Bai ZP, Wang ZL (2017). Combined use of land use regression and BenMAP for estimating public health benefits of reducing PM2.5 in Tianjin, China. Atmos Environ.

[CR4] Chen C, Zhu PF, Lan L, Zhou L, Liu RC, Sun QH, Ban J, Wang WT, Xu DD, Li TT (2018). Short-term exposures to PM2.5 and cause-specific mortality of cardiovascular health in China. Environ Res.

[CR5] Chen GB, Wang AX, Li SS, Zhao XQ, Wang YL, Li H, Meng X, Knibbs LD, Bell ML, Abramson MJ, Wang YJ, Guo YM (2019). Long-term exposure to air pollution and survival after ischemic stroke. Stroke.

[CR6] Conklin DJ, Schick S, Blaha MJ, Carll A, DeFillippis A, Ganz P, Hall ME, Hamburg N, O’Toole T, Reynolds L, Srivastava S, Bhatnagar A (2019). Cardiovascular injury induced by tobacco products: assessment of risk factors and biomarkers of harm. A Tobacco Centers of Regulatory Science compilation. Am J Physiol Heart Circ Physiol.

[CR7] Dai XT, Liu H, Chen DF, Zhang J (2018). Association between ambient particulate matter concentrations and hospitalization for ischemic heart disease (I20-I25, ICD-10) in China: a multicity case-crossover study. Atmos Environ.

[CR8] Devos S, Cox B, Dhondt S, Nawrot T, Putman K (2015). Cost saving potential in cardiovascular hospital costs due to reduction in air pollution. Sci Total Environ.

[CR9] Dockery DW, Pope CA, Xu X, Spengler JD, Ware JH, Fay ME (1993). An association between air pollution and mortality in six U.S. cities. N Engl J Med.

[CR10] Dong HB, Yu YQ, Yao S, Lu Y, Chen ZY, Li GY, Yao Y, Yao XJ, Wang SL, Zhang Z (2018). Acute effects of air pollution on ischaemic stroke onset and deaths: a time-series study in Changzhou, China. BMJ Open.

[CR11] GBD (2017). Risk factor collaborators (2018) global, regional, and national comparative risk assessment of 84 behavioural, environmental and occupational, and metabolic risks or clusters of risks for 195 countries and territories, 1990-2017: a systematic analysis for the global burden of disease study 2017. Lancet.

[CR12] Gouveia N, de Freitas CU, Martins LC, Marcilio IO (2006). Respiratory and cardiovascular hospitalizations associated with air pollution in the city of Sao Paulo, Brazil. Cadernos De Saúde Pública.

[CR13] Guan TJ, Xue T, Liu YL, Zheng YX, Fan SY, He KB, Zhang Q (2018). Differential susceptibility in ambient particle-related risk of first-ever stroke: findings from a national case-crossover study. Am J Epidemiol.

[CR14] Guo P, Wang YL, Feng WR, Wu JG, Fu CX, Deng H, Huang J, Wang L, Zheng MR, Liu HZ (2017). Ambient air pollution and risk for ischemic stroke: a short-term exposure assessment in South China. Int J Environ Res Public Health.

[CR15] Guo B, Chen F, Deng Y, Zhang HL, Qiao X, Qiao ZJ, Ji K, Zeng J, Luo B, Zhang W, Zhang YQ, Zhao X (2018). Using rush hour and daytime exposure indicators to estimate the short-term mortality effects of air pollution: a case study in the Sichuan Basin, China. Environ Pollut.

[CR16] Huang ZJ, Zhou YQ, Lu Y, Duan YZ, Tang XH, Deng QH, Yuan H (2017). A case-crossover study between fine particulate matter elemental composition and emergency admission with cardiovascular disease. Acta Cardiol Sin.

[CR17] Liang RM, Yin P, Wang LJ, Li YC, Zhou MG (2017). Acute effect of fine particulate matters on daily cardiovascular disease mortality in seven cities of China. Zhonghua Liu Xing Bing Xue Za Zhi.

[CR18] Lin HL, Tao J, Du YD, Liu T, Qian ZM, Tian LW (2016). Particle size and chemical constituents of ambient particulate pollution associated with cardiovascular mortality in Guangzhou, China. Environ Pollut.

[CR19] Lin HL, Tao J, Du YD, Liu T, Qian ZM, Tian LW (2016). Differentiating the effects of characteristics of PM pollution on mortality from ischemic and hemorrhagic strokes. Int J Hyg Environ Health.

[CR20] Lin HL, Liu T, Xiao JP, Zeng WL, Guo LC, Li X, Xu YJ, Zhang YH, Chang JJ, Vaughn MG, Qian JM, Ma WJ (2017). Hourly peak PM2.5 concentration associated with increased cardiovascular mortality in Guangzhou, China. J Expo Sci Environ Epidemiol.

[CR21] Lin HL, Wang XJ, Qian ZM, Guo S, Yao ZJ, Vaughn MG, Dong GH, Liu T, Xiao JP, Li X, Zeng WL, Xu YJ, Ma WJ (2018) Daily exceedance concentration hours: A novel indicator to measure acute cardiovascular effects of PM2.5 in six Chinese subtropical cities. Environmental Research 111:117–123. 10.1016/j.envint.2017.11.022 29190528

[CR22] Liu H, Tian YH, Xu Y, Zhang J (2017). Ambient particulate matter concentrations and hospitalization for stroke in 26 Chinese cities: a case-crossover study. Stroke.

[CR23] Liu MM, Huang YN, Jin Z, Ma ZW, Liu XY, Zhang B, Liu Y, Yu Y, Wang JN, Bi J, Kinney PL (2017). The nexus between urbanization and PM2.5 related mortality in China. Environ Pollut.

[CR24] Liu MY, Xue XX, Zhou BS, Zhang YW, Sun BJ, Chen JP, Li XL (2019). Population susceptibility differences and effects of air pollution on cardiovascular mortality: epidemiological evidence from a time-series study. Environ Sci Pollut Res Int.

[CR25] Lozano R, Naghavi M, Foreman K, Lim S, Shibuya K, Aboyans V, Abraham J, Adair T, Aggarwal R, Ahn SY, AlMazroa MA, Alvarado M, Anderson HR, Anderson LM, Andrews KG, Atkinson C, Baddour LM, Barker-Collo S, Bartels DH, Bell ML, Benjamin EJ, Bennett D, Bhalla K, Bikbov B, Abdulhak AB, Birbeck G, Blyth F, Bolliger I, Boufous S, Bucello C, Burch M, Burney P, Carapetis J, Chen H, Chou D, Chugh SS, Coffeng LE, Colan SD, Colquhoun S, Colson KE, Condon J, Connor MD, Cooper LT, Corriere M, Cortinovis M, de Vaccaro KC, Couser W, Cowie BC, Criqui MH, Cross M, Dabhadkar KC, Dahodwala N, de Leo D, Degenhardt L, Delossantos A, Denenberg J, Des Jarlais DC, Dharmaratne SD, Dorsey ER, Driscoll T, Duber H, Ebel B, Erwin PJ, Espindola P, Ezzati M, Feigin V, Flaxman AD, Forouzanfar MH, Fowkes FGR, Franklin R, Fransen M, Freeman MK, Gabriel SE, Gakidou E, Gaspari F, Gillum RF, Gonzalez-Medina D, Halasa YA, Haring D, Harrison JE, Havmoeller R, Hay RJ, Hoen B, Hotez PJ, Hoy D, Jacobsen KH, James SL, Jasrasaria R, Jayaraman S, Johns N, Karthikeyan G, Kassebaum N, Keren A, Khoo JP, Knowlton LM, Kobusingye O, Koranteng A, Krishnamurthi R, Lipnick M, Lipshultz SE, Ohno SL, Mabweijano J, MacIntyre MF, Mallinger L, March L, Marks GB, Marks R, Matsumori A, Matzopoulos R, Mayosi BM, McAnulty JH, McDermott MM, McGrath J, Memish ZA, Mensah GA, Merriman TR, Michaud C, Miller M, Miller TR, Mock C, Mocumbi AO, Mokdad AA, Moran A, Mulholland K, Nair MN, Naldi L, Narayan KMV, Nasseri K, Norman P, O’Donnell M, Omer SB, Ortblad K, Osborne R, Ozgediz D, Pahari B, Pandian JD, Rivero AP, Padilla RP, Perez-Ruiz F, Perico N, Phillips D, Pierce K, Pope CA, Porrini E, Pourmalek F, Raju M, Ranganathan D, Rehm JT, Rein DB, Remuzzi G, Rivara FP, Roberts T, de León FR, Rosenfeld LC, Rushton L, Sacco RL, Salomon JA, Sampson U, Sanman E, Schwebel DC, Segui-Gomez M, Shepard DS, Singh D, Singleton J, Sliwa K, Smith E, Steer A, Taylor JA, Thomas B, Tleyjeh IM, Towbin JA, Truelsen T, Undurraga EA, Venketasubramanian N, Vijayakumar L, Vos T, Wagner GR, Wang M, Wang W, Watt K, Weinstock MA, Weintraub R, Wilkinson JD, Woolf AD, Wulf S, Yeh PH, Yip P, Zabetian A, Zheng ZJ, Lopez AD, Murray CJL (2012). Global and regional mortality from 235 causes of death for 20 age groups in 1990 and 2010: a systematic analysis for the global burden of disease study 2010. Lancet.

[CR26] Mills NL, Donaldson K, Hadoke PW, Boon NA, MacNee W, Cassee FR, Sandstrom T, Blomberg A, Newby DE (2009). Adverse cardiovascular effects of air pollution. Nat Clin Pract Cardiovasc Med.

[CR27] Mozaffarian D, Benjamin EJ, Go AS, Arnett DK, Blaha MJ, Cushman M (2016). Heart disease and stroke Statistics-2016 update: a report from the American Heart Association. Circulation.

[CR28] National Center for Cardiovascular Diseases (2017). Report on cardiovascular diseases in China 2016 (Chinese).

[CR29] Newby DE, Mannucci PM, Tell GS, Baccarelli AA, Brook RD, Donaldson K, Forastiere F, Franchini M, Franco OH, Graham I, Hoek G, Hoffmann B, Hoylaerts MF, Kunzli N, Mills N, Pekkanen J, Peters A, Piepoli MF, Rajagopalan S, Storey RF (2014). Expert position paper on air pollution and cardiovascular disease. Eur Heart J.

[CR30] Pan X, Gong YY, Martinelli I, Angelici L, Favero C, Bertazzi PA, Mannucci PM, Ariens RAS, Routledge MN (2016). Fibrin clot structure is affected by levels of particulate air pollution exposure in patients with venous thrombosis. Environ Int.

[CR31] Pope CA, Burnett RT, Thurston GD, Thun MJ, Calle EE, Krewski D, Godleski JJ (2004). Cardiovascular mortality and long-term exposure to particulate air pollution: epidemiological evidence of general pathophysiological pathways of disease. Circulation.

[CR32] Qiu H, Yu ITS, Wang XR, Tian LW, Tse LA, Wong TW (2013). Differential effects of fine and coarse particles on daily emergency cardiovascular hospitalizations in Hong Kong. Atmos Environ.

[CR33] Qiu H, Sun SZ, Tsang H, Wong CM, Lee RSY, Schooling CM, Tian LW (2017). Fine particulate matter exposure and incidence of stroke: a cohort study in Hong Kong. Neurology.

[CR34] Rajagopalan S, Al-Kindi SG, Brook RD (2018). Air pollution and cardiovascular disease JACC state-of-the-art review. J Am Coll Cardiol.

[CR35] Requia WJ, Adams MD, Arain A, Papatheodorou S, Koutrakis P, Mahmoud M (2018). Global association of air pollution and cardiorespiratory diseases: a systematic review, meta-analysis, and investigation of modifier variables. Am J Public Health.

[CR36] Robertson S, Miller MR (2018). Ambient pollution and thrombosis. Part Fibre Toxicol.

[CR37] Sheehan P, Cheng EJ, English A, Sun FH (2014). China’s response to the air pollution shock. Nat Clim Chang.

[CR38] Su C, Breitner S, Schneider A, Liu LQ, Franck U, Peters A, Pan XC (2016). Short-term effects of fine particulate air pollution on cardiovascular hospital emergency room visits: a time-series study in Beijing, China. Int Arch Occup Environ Health.

[CR39] Tian YH, Xiang X, Wu YQ, Cao YY, Song J, Sun KX, Liu H, Hu YH (2017). Fine particulate air pollution and first hospital admissions for ischemic stroke in Beijing, China. Sci Rep.

[CR40] Tian YH, Liu H, Zhao ZL, Xiang X, Li M, Juan J, Song J, Cao YY, Wang XW, Chen LB, Wei C, Hu YH, Gao P (2018). Association between ambient air pollution and daily hospital admissions for ischemic stroke: a nationwide time-series analysis. PLoS Med.

[CR41] Tong YR, Luo K, Li RK, Pei L, Li A, Yang MG, Xu Q (2018). Association between multi-pollutant mixtures pollution and daily cardiovascular mortality: an exploration of exposure-response relationship. Atmos Environ.

[CR42] Wang XD, Zhang XM, Zhuang SW, Luo Y, Kang S, Liu YL (2016). Short-term effects of air pollution on acute myocardial infarctions in Shanghai, China, 2013–2014. J Geriatr Cardiol.

[CR43] Wang JF, Yin Q, Tong SL, Ren ZP, Hu MG, Zhang HR (2017). Prolonged continuous exposure to high fine particulate matter associated with cardiovascular and respiratory disease mortality in Beijing, China. Atmos Environ.

[CR44] Wang XJ, Qian ZM, Wang XJ, Hong H, Yang Y, Xu YJ, Xu XJ, Yao ZJ, Zhang LL, Rolling CA, Schootman M, Liu T, Xiao JP, Li X, Zeng WL, Ma WJ, Lin HL (2018). Estimating the acute effects of fine and coarse particle pollution on stroke mortality of in six Chinese subtropical cities. Environ Pollut.

[CR45] Wu TT, Ma Y, Wu X, Bai M, Peng Y, Cai WT, Wang YX, Zhao J, Zhang Z (2019). Association between particulate matter air pollution and cardiovascular disease mortality in Lanzhou, China. Environ Sci Pollut Res.

[CR46] Xia R, Zhou GP, Zhu T, Li XY, Wang GF (2017). Ambient air pollution and out-of-hospital cardiac arrest in Beijing, China. Int J Environ Res Public Health.

[CR47] Xie WX, Li G, Zhao D, Xie XQ, Wei ZH, Wang W, Wang M, Li GX, Liu WR, Sun JY, Jia ZR, Zhang Q, Liu J (2015). Relationship between fine particulate air pollution and ischaemic heart disease morbidity and mortality. Heart.

[CR48] Xu AY, Mu Z, Jiang B, Wang W, Yu H, Zhang LZ, Li J (2017). Acute effects of particulate air pollution on ischemic heart disease hospitalizations in Shanghai, China. Int J Environ Res Public Health.

[CR49] Xu Q, Wang S, Guo YM, Wang C, Huang FF, Li X, Gao Q, Wu LJ, Tao LX, Guo J, Wang W, Guo XH (2017). Acute exposure to fine particulate matter and cardiovascular hospital emergency room visits in Beijing, China. Environ Pollut.

[CR50] Ye RZ, Cui LL, Peng XM, Yu KK, Cheng F, Zhu YK, Jia CQ (2019). Effect and threshold of PM2.5 on population mortality in a highly polluted area: a study on applicability of standards. Environ Sci Pollut Res Int.

[CR51] Yin P, Brauer M, Cohen A, Burnett RT, Liu JM, Liu YN, Liang RM, Wang WH, Qi JL, Wang LJ, Zhou MG (2017). Long-term fine particulate matter exposure and nonaccidental and cause-specific mortality in a large national cohort of Chinese men. Environ Health Perspect.

[CR52] Yu YQ, Yao S, Dong HB, Ji MH, Chen ZY, Li GY, Yao XJ, Wang SL, Zhang Z (2018). Short-term effects of ambient air pollutants and myocardial infarction in Changzhou, China. Environ Sci Pollut Res.

[CR53] Yuan S, Wang JX, Jiang QQ, He ZY, Huang YC, Li ZY, Cai LY, Cao SY (2019). Long-term exposure to PM2.5 and stroke: a systematic review and meta-analysis of cohort studies. Environ Res.

[CR54] Zang X, Qi X (2017). Association between PM 2.5 exposure and the prognosis of patients with acute myocardial infarction. Arch Med Res.

[CR55] Zhang Q, Qi WP, Yao W, Wang M, Chen YY, Zhou YJ (2016). Ambient particulate matter (PM_2.5_/PM_10_) exposure and emergency department visits for acute myocardial infarction in Chaoyang District, Beijing, China during 2014: a case-crossover study. J Epidemiol.

[CR56] Zhang C, Quan ZY, Wu QC, Jin ZZ, Lee JH, Li CH, Zheng YX (2018). Association between atmospheric particulate pollutants and mortality for cardio-cerebrovascular diseases in Chinese Korean population: a case-crossover study. Int J Environ Res Public Health.

[CR57] Zhang RH, Liu GF, Jiang Y, Li G, Pan YS, Wang YL, Wei ZH, Wang J, Wang YJ (2018). Acute effects of particulate air pollution on ischemic stroke and hemorrhagic stroke mortality. Front Neurol.

[CR58] Zheng PW, Shen P, Ye ZH, Zhang ZY, Chai PF, Li D, Jin MJ, Tang ML, Lu HC, Lin HB, Wang JB, Chen K (2018). Acute effect of fine and coarse particular matter on cardiovascular visits in Ningbo, China. Environ Sci Pollut Res.

